# A qualitative evaluation of team and family perceptions of family-based treatment delivered by videoconferencing (FBT-V) for adolescent Anorexia Nervosa during the COVID-19 pandemic

**DOI:** 10.1186/s40337-022-00631-9

**Published:** 2022-07-26

**Authors:** Jennifer Couturier, Danielle Pellegrini, Laura Grennan, Maria Nicula, Catherine Miller, Paul Agar, Cheryl Webb, Kristen Anderson, Melanie Barwick, Gina Dimitropoulos, Sheri Findlay, Melissa Kimber, Gail McVey, Rob Paularinne, Aylee Nelson, Karen DeGagne, Kerry Bourret, Shelley Restall, Jodi Rosner, Kim Hewitt-McVicker, Jessica Pereira, Martha McLeod, Caitlin Shipley, Sherri Miller, Ahmed Boachie, Marla Engelberg, Samantha Martin, Jennifer Holmes-Haronitis, James Lock

**Affiliations:** 1 McMaster University, Hamilton, ON Canada; 2 McMaster Children’s Hospital, 1200 Main St W, Hamilton, ON L8N 3Z5 Canada; 3 Canadian Mental Health Association – Waterloo Wellington, Kitchener, ON Canada; 4 Chicago Center for Evidence-Based Treatment, Chicago, IL USA; 5 University of Toronto, Toronto, ON Canada; 6 Research Institute, The Hospital for Sick Children, Toronto, ON Canada; 7 University of Calgary, Calgary, AB Canada; 8 University Health Network, Toronto, ON Canada; 9 St. Joseph’s Care Group, Thunder Bay, ON Canada; 10 Grand River Hospital, Kitchener, ON Canada; 11 Southlake Regional Health Centre, Newmarket, ON Canada; 12 North York General Hospital, North York, ON Canada; 13 Stanford University, Stanford, CA USA

**Keywords:** Eating disorders, Anorexia Nervosa, Virtual care, Family-based treatment, COVID-19, Therapists, Medical practitioners, Program administrators, Families, Qualitative research

## Abstract

**Background:**

During the COVID-19 pandemic, outpatient eating disorder care, including Family-Based Treatment (FBT), rapidly transitioned from in-person to virtual delivery in many programs. This paper reports on the experiences of teams and families with FBT delivered by videoconferencing (FBT-V) who were part of a larger implementation study.

**Methods:**

Four pediatric eating disorder programs in Ontario, Canada, including their therapists (n = 8), medical practitioners (n = 4), administrators (n = 6), and families (n = 5), participated in our study. We provided FBT-V training and delivered clinical consultation. Therapists recorded and submitted their first four FBT-V sessions. Focus groups were conducted with teams and families at each site after the first four FBT-V sessions. Focus group transcripts were transcribed verbatim and key concepts were identified through line-by-line reading and categorizing of the text. All transcripts were double-coded. Focus group data were analyzed using directed and summative qualitative content analysis.

**Results:**

Analysis of focus group data from teams and families revealed four overarching categories—pros of FBT-V, cons of FBT-V, FBT-V process, and suggestions for enhancing and improving FBT-V. Pros included being able to treat more patients and developing a better understanding of family dynamics by being virtually invited into the family’s home (identified by teams), as well as convenience and comfort (identified by families). Both teams and families recognized technical difficulties as a potential con of FBT-V, yet teams also commented on distractions in family homes as a con, while families expressed difficulties in developing therapeutic rapport. Regarding FBT-V process, teams and families discussed the importance and challenge of patient weighing at home. In terms of suggestions for improvement, teams proposed assessing a family’s suitability or motivation for FBT-V to ensure it would be appropriate, while families strongly suggested implementing hybrid models of FBT in the future which would include some in-person and some virtual sessions.

**Conclusion:**

Team and family perceptions of FBT-V were generally positive, indicating acceptability and feasibility of this treatment. Suggestions for improved FBT-V practices were made by both groups, and require future investigation, such as examining hybrid models of FBT that involve in-person and virtual elements.

*Trial registration* ClinicalTrials.gov NCT04678843.

**Supplementary Information:**

The online version contains supplementary material available at 10.1186/s40337-022-00631-9.

## Plain English summary

Family-Based Treatment—the leading outpatient treatment for children and adolescents with eating disorders—rapidly transitioned to virtual delivery during the COVID-19 pandemic in many programs. This study qualitatively describes the perceptions and experiences of teams and families related to the first four sessions of Family-Based Treatment delivered via videoconferencing (FBT-V) during the COVID-19 pandemic, using end-of-study focus groups. Our findings suggest that FBT-V is generally well-received amongst these stakeholders. Therapists found that implementing FBT-V was relatively seamless, and families described the convenience and comfort of sessions taking place virtually in their own homes. However, therapists revealed concerns of distractions in family homes impacting sessions, and families had some difficulties in developing therapeutic rapport. As a result, additional modes of delivering Family-Based Treatment should be explored to address challenges present in virtual care. This might include hybrid models of treatment delivery, involving a combination of in-person and virtual sessions.

**Supplementary Information:**

The online version contains supplementary material available at 10.1186/s40337-022-00631-9.

## Introduction

It is well known that the COVID-19 pandemic has had a significant detrimental impact on those affected by and treating eating disorders [1, 2]. In addition to a worsening of eating disorder-related symptoms, individuals with eating disorders have also reported higher levels of stress, anxiety, depression, and post-traumatic stress disorder symptoms throughout the pandemic [1]. Some healthcare providers treating eating disorder patients have also experienced a sense of therapeutic inefficiency and compromised therapeutic alliance [2], as they struggle to manage the surge in eating disorder cases, emergency department visits, and hospital admissions—particularly among youth—during this time [3–6].

The most widely used evidence-based treatment for children and adolescents with eating disorders is Family-Based Treatment (FBT) [7–9], in which parents are placed in charge of the refeeding process and interrupting disordered eating behaviours, while being supported by a therapist during family sessions [8]. The term FBT is often used interchangeably with Family Therapy for Anorexia Nervosa (FT-AN) and Maudsley Family Therapy, as they all refer to eating disorder focused family therapy following the same principles and shared theoretical frameworks, however FBT differs slightly in that a published treatment manual exists for FBT [10].

Although there is a growing body of evidence supporting the clinical effectiveness of FBT adapted for virtual care, uncertainties remain with respect to its implementation, particularly during the ongoing COVID-19 pandemic; research in virtual adaptations of FBT are currently limited to case studies and small sample feasibility studies [11, 12].

With COVID-19 variants continuing to emerge, it is anticipated that virtual care will be regularly offered in the foreseeable future [13]. While convenient, the nature of virtual treatment adaptations and their effective delivery requires examination to ensure acceptability and feasibility among those delivering and those receiving the treatment. Adaptations that focus on improving treatment suitability for the target population can lead to improved engagement, acceptability, and clinical outcomes [14], but modifications that alter or remove core components of a treatment, or which neglect population needs, may be less accepted among patient or provider populations [15]. Failing to understand the treatment modifications needed for virtual care, as well as the systematic processes that can contribute to implementation or success, may hinder outcomes, acceptability, and feasibility [16, 17].

Past research suggests that virtual models and adaptations of therapy are acceptable and feasible, including FBT [11, 18]. In a study that explored the acceptability and feasibility of delivering FBT via telehealth, all 10 adolescent participants remained in treatment after 20 sessions over six months, suggesting acceptability and feasibility, and their percent mean body mass index significantly increased at post-intervention and 6-month follow-up [11]. A recent case study during the COVID-19 pandemic, including a young adult and an adolescent, examined a virtual adaptation of FBT delivered over a 4-week period. Adaptations involved an enhanced multidisciplinary team (e.g., family therapist, dietician, peer mentor, and family mentor) and virtual treatment delivery; findings indicated strong acceptability among the two patients. In addition, both patients achieved the desired weight gain and a reduction in eating disorder symptoms [18]. Additionally, experts in FBT have commented on delivering FBT via videoconferencing during the COVID-19 pandemic, noting the many challenges of transitioning to virtual care in this field, and highlighting the need for further examination of acceptability and feasibility of this treatment [19].

Our study evaluated the initial implementation of FBT delivered by videoconferencing (FBT-V) in four pediatric eating disorder treatment programs in Ontario, Canada. We hoped to further develop clinical capacity for virtual care within our healthcare system for this population, and to improve access to evidence-based pediatric eating disorder treatment during the COVID-19 pandemic and beyond. This paper reports on the qualitative findings of the study, specifically examining the experience of delivering and receiving the first four sessions of FBT-V from the perspectives of teams and families.

## Method

This paper reports on the qualitative experiences of teams and families involved in a larger FBT-V implementation study conducted in Ontario, Canada. Ethical approval was received from the Hamilton Integrated Research Ethics Board, as well as the ethics boards/committees at each of the participating sites. Details pertaining to the objectives and methodology of the larger study are published within a protocol paper [20]. Findings pertaining to our implementation approach which comment on fidelity, maintenance of key components of FBT, and patient outcomes have been submitted for publication elsewhere [21].

For the current qualitative study, the principles of qualitative description [22] were followed and our research team completed semi-structured focus groups with teams and families who had provided or received FBT-V during the COVID-19 pandemic within our implementation study. Teams included therapists, medical practitioners and administrators at each site. Although the whole team participated in their respective focus group, the therapist was most able to speak to the delivery of FBT-V and thus, team qualitative data in this paper mostly arises from the therapists. Despite this, medical practitioners and program administrators were included in the focus groups as they participated in the FBT-V training and implementation consultation meetings in our study. By allowing these individuals the opportunity to comment on FBT-V and our implementation approach, this further expanded our understanding of what FBT-V implementation entails in an eating disorder program. As program administrators are responsible for managing the program, and medical practitioners diagnose the patient with an eating disorder, it is important that they are on board with supporting FBT-V, to ensure the best possible care is provided to patients. As a result, it is important to capture medical practitioner and program administrator thoughts on the implementation of FBT-V, in addition to those of therapists.

### Setting and participants

As indicated in our larger FBT-V implementation study [20], four pediatric eating disorder programs in Ontario, Canada participated in our study. These sites vary in terms of geography and service capacity, as two are located in large urban areas and are based in hospitals, offering inpatient, day hospital, and outpatient services for eating disorders, whereas the other two sites are community-based organizations located in smaller regions that can only provide outpatient eating disorder services.

Eighteen individuals from the four sites and five families (n = 21) were recruited for this project (see Table 1 for detailed demographic information). In order to participate, therapists had to have had prior training and experience in delivering standard FBT. Of the eight participating therapists, only five were able to successfully recruit a patient for the study; the other three therapists remained in the study and continued to participate in consultation meetings to learn from their colleagues, as well as the end-of-study focus group. This allowed them to provide their thoughts on FBT-V, given that they continued to deliver FBT-V despite not having a study patient, as well as having participated in other aspects of our implementation approach, including the FBT-V training workshop, and therefore they should be given the opportunity to comment on this. All participating patients were diagnosed with Anorexia Nervosa by the medical practitioners involved in the study. Two participants (one medical practitioner and one therapist) withdrew from the study due to medical absences from work that were unrelated to the study; another participant (one administrator) withdrew from the study due to retirement.

**Table 1:** Demographic characteristics of study participants

Characteristics	Number of participants	Range (years)	Mean ± SD (years)
*Therapists, medical practitioners, administrators* (n = 18)			
Site			
Site 1	4		
Site 2	3		
Site 3	5		
Site 4	6		
Age		31–66	45.11 ± 7.83
Gender			
Female	17		
Male	1		
Role			
Therapist	8		
Medical practitioner (MD or NP)	4		
Administrator	6		
Years in current role		2–20	10.11 ± 6.57
*Families (n = 5)*			
Site			
Site 1	1		
Site 2	1		
Site 3	2		
Site 4	1		
Patient age		13–16	14.40 ± 1.14
Patient gender			
Female	4		
Male	1		
Family members			
Patient	5		
Parent/caregiver	10		
Sibling	6		

SD, standard deviation; MD, medical doctor; NP, nurse practitioner

### Clinical training and consultation

All participating staff from all four sites attended a virtual, half-day training workshop for FBT-V, which was to be used as a guide for implementing FBT-V in this study. The training workshop specifically reviewed the virtual aspects of FBT-V implementation, including key components of FBT to be maintained in virtual delivery, fidelity to the FBT-V model, and potential barriers to success; an opportunity to openly discuss experiences of virtual care to date, especially throughout the pandemic, was also provided during the workshop. Training was led by external experts (JL and KA) and local experts (JC and CW) on the study team. After training, each therapist was invited to enroll one eligible family (adolescent < 18 years of age with Anorexia Nervosa) into FBT-V and video record their first four sessions, each of which were submitted to the research team for review. Bi-weekly group clinical consultation video calls were provided for therapists, respectively with each site.

### Intervention

FBT is the leading outpatient treatment for children and adolescents with eating disorders [7–9]. This manualized treatment utilizes the family as the primary resource to renourish the affected individual [8]. It involves approximately 9–12 months of regular treatment that decreases in frequency over time with one therapist guiding each family session and a physician overseeing the physical health of the child. Our study intervention involved a virtual adaption of FBT (FBT-V), where therapy sessions occurred virtually via Zoom for Healthcare, but medical appointments for the child remained in-person. For a full description of the adaptations made to FBT for virtual delivery please see our protocol paper [20]. Therapists were instructed to conduct FBT-V sessions following the same key components and principles of FBT, as outlined in the FBT manual [8]. This included obtaining patient weights (either with the therapist present on screen with only the child, or the parent(s) weighing the child in session with the therapist present—both acceptable) and reviewing the weight graph with the family, providing an opportunity to independently speak with the child (e.g., at the beginning of the session before or after weighing of the patient), and conducting a family meal in session 2, all while using externalization and agnosticism throughout.

### Data collection

Semi-structured focus groups were used to explore team experiences with FBT-V delivery. Semi-structured focus groups were also used to examine family (patient, parent(s) and sibling(s)—if any) experiences with FBT-V. Focus groups were conducted virtually at the end of the study (after four FBT-V sessions for the families, or after all therapists had completed four FBT-V sessions at each site for the teams) and were video recorded and transcribed verbatim for data analysis. The creation of focus group guides and interviewing style were informed by the principles of qualitative description [22]. See Additional files 1 and 2 for the focus group guides used in this study.

### Qualitative methods and analysis

To generate a description and understanding of the participants’ perceptions and experiences of FBT-V, we used directed and summative qualitative content analysis to analyze focus group data [23]. While inductive approaches are often used to generate new theories or insights from a lack of existing research, the deductive approach of directed content analysis is used when prior research exists about the phenomenon of interest [23]. We chose a deductive approach based upon existing literature about virtual implementation of eating disorder treatment for pediatric patients, including FBT [11, 12]. Focus group transcript data were allocated to codes, and any text that did not fit into the initial coding scheme was provided a new code. Codes were continuously refined through multiple readings of the transcripts and in consultation with the research team, until all codes were appropriate and applied accurately. Finally, data that fit under each code were represented as counts through summative content analysis, while also presenting representative quotes to contextualize these findings. Compared to other qualitative analytics techniques, content analysis was chosen as we wanted to stay close to the data and not make interpretations; a summative content analysis was chosen to provide counts of codes and because it also introduces minimal interpretation into analysis while offering a “straight description” of the patterns or regularities of participants responses [22, 24]. All transcripts were coded in duplicate by two co-authors. A third team member resolved any coding conflicts via the facilitation of a consensus meeting with the two coders. Qualitative data and coding procedures were managed using NVivo 10 (QSR International Pty Ltd., Version 8, 2008).

## Results

### Team perceptions

For a summative description of team-specific data, see Table 2. As indicated above, almost all perceptions related to FBT-V and its delivery were from therapists. Although therapists generally reported more advantages than disadvantages in delivering FBT-V, it was evident that therapists thought there was room for improvement, particularly in ensuring FBT-V is suitable for the family, and not solely implemented out of convenience for all parties involved.

**Table 2:** Categories and subcategories emerging from qualitative analysis among teams (n = 18 participants; 4 focus groups)

Category	Subcategory	Frequency
Pros of FBT-V	Easy to use and deliver treatment	3 Participants, 3 references
	Effective	2 Participants, 2 references
	Having the ability to treat more patients	1 Participant, 1 reference
	Developing a better understanding of family dynamics	1 Participant, 1 reference
Cons of FBT-V	Technical difficulties	2 Participants, 2 references
	Lack of commitment to and preparation for sessions by families	1 Participant, 1 reference
	Distractions in the family home	1 Participant, 2 references
FBT-V process	Independent time between therapist and patient is important	1 Participant, 1 reference
	Obtaining and showing patient weights during sessions and facilitating discussions about weight changes is important	2 Participants, 2 references
	Medical practitioner role did not change in FBT-V	2 Participants, 2 references
Suggestions for improvement	Recording and sharing video recorded FBT-V sessions with families	3 Participants, 3 references
	Having family members log into the virtual platform on different devices	1 Participant, 1 reference
	Assessing suitability and motivation for FBT-V among each family	1 Participant, 1 reference
	To continue offering FBT-V	1 Participant, 1 reference

#### Pros of FBT-V

Therapists found that once they became familiar and comfortable with their virtual platform, implementing FBT-V was relatively seamless, especially for those who had been delivering virtual therapy prior to the pandemic. Moreover, therapists believed that compared to in-person FBT, FBT-V can be as effective as standard (i.e., in-person) FBT care. One therapist stated:

“*I was really pleasantly surprised at how effective treatment could be virtually because prior to the pandemic, I had no experience delivering therapy over a virtual format. So, I was actually really pleased with how well it went.*” (Site 3).

Therapists described several benefits associated with this virtual treatment. For example, being able to treat more patients, especially those who might have had trouble accessing in-person treatment if they reside far away from clinics/hospitals, was described as a benefit by one therapist. It was also reported that having the ability to witness family dynamics in their own homes, rather than in an office setting, allowed therapists to obtain a better understanding of the family dynamic and challenges. One therapist said:

“*…when you’re being invited into somebody’s home* [during a FBT-V session], *there’s a lot of really strong positives… if we’re comparing how to do it in the office versus how to do it online, there’s a lot of benefits to be able to do a family meal, for example, and watch the family as they interact in real life in their family home.*” (Site 1).

#### Cons of FBT-V

Although the consensus regarding FBT-V was positive, some cons to virtual delivery of FBT were identified. Therapists remarked that technical difficulties threatened the impact of sessions, particularly for families located in remote settings and/or with poor internet connections. Therapists did not believe that technical difficulties in this study significantly impacted any of their sessions, however they were described as frustrating when they did occur. Additionally, one therapist described lower levels of commitment to and preparation for sessions amongst some families receiving FBT-V, in comparison to the preparation that typically precedes in-person treatment (e.g., having to drive to an appointment versus logging onto a computer several minutes before a session). This therapist stated that this lack of physical and mental preparation and anticipation before a session could contribute to reduced motivation for treatment and less impactful sessions, which could pose a risk to treatment success. The same therapist also recognized that while it can be beneficial to be welcomed into the homes of their patients virtually through FBT-V, this can also be unfavourable, as therapists may have less control over FBT-V sessions due to unexpected distractions occurring in the family homes, which can detract from serious discussions:

“…*When you’re going into somebody’s house, you have to respect their house… there is something different about going into somebody’s home… you’re on their turf versus them being on your turf… in* [their] *house when a kid might leave the room or somebody knocks at the door for a package and all of a sudden you’ve got a disruption… You don’t have control over that if it does happen. You do have more control over that in your office. We’re here for a specific time...There aren’t going to be any distractions…*” (Site 1).

#### FBT-V process

There was some dialogue that reinforced the importance of therapists incorporating private time between themselves and the adolescent (without the family) in each session, as well as parents obtaining and showing patient weights in FBT-V and facilitating discussion about any influences on weight changes of the patient with the family. One therapist explained that while they had not been showing the patient weights to the patient and their family in FBT-V in their private practice, witnessing the impact this had with their patient in this study has since sparked a change in their practice:

*“I’ve started* [showing patient weights during FBT-V sessions] *because of this* [study]*, and I’m finding it really, really useful.”* (Site 3).

Additionally, given that medical practitioners continued to see study patients in-person in this study, they felt that their role did not change in FBT-V. One medical practitioner expanded on this, saying:

“*No, I think it probably if anything* [the study]* just reinforced my role a little bit more clearly. But I wouldn’t say I changed dramatically between patients in the study versus those not. Maybe I’m a bit more aware, but no, there wasn’t a lot of change for me.*” (Site 4).

#### Suggestions for improvement

Teams also discussed suggestions for improvement to future FBT-V practice. This included proposing the option to families to video record FBT-V sessions and sharing video recordings with the families at later stages of treatment, to demonstrate their progress, as indicated by the members of one implementation team. One therapist stated:

*“I almost wonder if* [sharing video recordings of their past FBT-V sessions with the family] *would be appropriate to do more at the end of phase two where people are kind of… they’ve turned a corner…Or even say six sessions in when there’s some weight gain and parents are able to externalize the eating disorder and looking back and saying, ‘okay, do you see the difference between the way your child presented?”* (Site 2).

Other therapists suggested ways to improve engagement with families, such as having some family members log into the virtual platform for treatment on different devices (e.g., patient/siblings on one screen and parents on another). One therapist believed that this might be beneficial to hear all family members more clearly through virtual platforms, especially if working with large families or those with poor internet connections. Another suggestion included assessing a family’s suitability or motivation for FBT-V; the convenience and ease of logging into a virtual platform for treatment may enable some families to fall into a treatment that may not be the best fit for them. For this, emphasizing the expectations of virtual treatment ahead of time might help mitigate this issue.

Given the uncertainty of the COVID-19 pandemic, teams were unsure whether the delivery of FBT would ever return to only being offered in-person. However, one administrator from a remote Northern Ontario study site (i.e., the only centre offering pediatric eating disorder outpatient treatment in this region) commented that their program had been offering virtual treatment regularly prior to the pandemic, due to geographical barriers that commonly impede patients and their families from attending in-person visits. As a result, their site plans to continue offering and advocating for FBT-V beyond the pandemic, as they strongly believe it enables the best service and treatment for their patients in this region. This administrator said:

*“We have worked using virtual options for quite some time…and I’ve really pushed that because service our clients* [with virtual treatment options] *maybe much earlier than many other programs because of necessity. We just…absolutely needed to do this.”* (Site 1).

### Family perceptions

For a summative description of family-specific data, see Table 3. Almost all family participants reported a preference for in-person treatment instead of virtual care or a hybrid model, even if they had never previously received any type of in-person therapy. Only one individual (a parent) suggested that virtual care may be preferred, specifically if an individual is dealing with social anxiety and is not able to leave their home.

**Table 3:** Categories and subcategories emerging from qualitative analysis among families (n = 21 family members; 5 focus groups)

Category	Subcategory	Frequency
Pros of FBT-V	Convenience	8 Participants, 12 references
	Comfort	6 Participants, 9 references
	Cost-effectiveness	8 Participants, 8 references
	Virtual platform used in this study	7 Participants, 5 references
	Ease of use for children	3 Participants, 5 references
Cons of FBT-V	Technical difficulties	7 Participants, 13 references
	Trouble building a connection with therapist	7 Participants, 13 references
	Feeling anxious	6 Participants, 9 references
	Lack of familiarity with the virtual format	3 Participants, 3 references
FBT-V process	Family meal	5 Participants, 7 references
	Patient weighing	8 Participants, 9 references
	Impact on weight gain and eating disorder symptoms	10 Participants, 12 references
	Repeated reminders by therapists	4 Participants, 6 references
	Increased knowledge about eating disorders	4 Participants, 6 references
	Treatment focus	4 Participants, 4 references
	Improved family dynamics/communication	8 Participants, 11 references
	Inclusion of siblings	1 Participant, 1 reference
	Recommendation of FBT to another family	11 Participants, 11 references
Suggestions for improvement	Hybrid models of FBT	9 Participants, 17 references
	Patient choosing virtual or in-person treatment	1 Participant, 2 references
	Using alternative virtual platforms	1 Participant, 1 reference
	Increased one-on-one time between patient and therapist	5 Participants, 7 references
	Adding a second family meal session	2 Participants, 2 references
	Reducing session frequency	1 Participant, 1 reference

#### Pros of FBT-V

Despite the overwhelming support for in-person over virtual treatment, families described a variety of advantages to participating in virtually delivered care in this study, with the most common being convenience and comfort. For convenience, this included the ability to partake in treatment from home and not having to travel to sessions, evidenced by one participant stating:

*“I think it was great, honestly, to get all of us somewhere, another location, would’ve been more challenging, and* [whether] *we would even be able to do it would be a question...it was great having* [treatment] *right here on our dining room table.”* (Site 3).

In terms of comfort of FBT-V sessions, this was often referred to as being able to have difficult and emotional conversations in a familiar and relaxed setting, such as their family home:

“*I would say I felt more comfortable having conversations that are tough in our own home.*”

“*Yeah. It is definitely more comfortable in your own home.*” (Site 2).

Other benefits of virtual delivery of FBT reported by families included: (a) cost-effectiveness, as parents reported few costs incurred (e.g., time) in order to attend the sessions; (b) the virtual platform used in this study, which was reported as satisfactory for treatment (by parents and adolescents); and (c) the ease of use for children to adapt to a virtual format, as parents found that their children’s recent experiences with virtual school allowed them to adapt well to online therapy such as FBT-V.

#### Cons of FBT-V

Families also described some disadvantages to participating in FBT-V, namely technical difficulties and trouble building a connection with their therapist virtually. Regarding technical difficulties, one family regularly experienced glitches and freezing during their sessions:

*“That’s probably another thing that was a downside, was the technology all the time having* [difficulties]*…” “You’d talk over each other and…” “Say the same thing twice and…” “Again, the technical difficulties.”* (Site 2).

Additionally, families often expressed challenges in developing rapport with their therapist, specifically due to the virtual nature of their sessions, and feeling uncomfortable disclosing sensitive and personal content to someone that they have only met virtually. One parent stated:

*“I found it was a bit more challenging because we’ve never met* [therapist]*… I found that a bit difficult… I probably won’t do virtual* [again] *unless it was someone I really knew for a long time and I felt comfortable enough...I would have been maybe a bit more open or forthcoming if I had known her. Like in-person. I might have said different things or shared more, if I knew* [therapist] *or if it was in-person* [instead of] *on* [Zoom]*.”* (Site 4).

Other disadvantages of virtual delivery of FBT included feeling anxious in a virtual versus in-person setting, generally reported by children, as well as lack of familiarity with the virtual format (i.e., being more familiar with Microsoft Teams instead of Zoom for Healthcare), which was generally reported by parents.

#### FBT-V process

Acceptability of FBT-V was apparent amongst most family members. Families divulged their thoughts towards specific virtual adaptations, including the virtually delivered family meal and patient weighing conducted by the parents (as opposed to a clinician in standard FBT practice). Families typically reported the family meal session to be most uncomfortable regardless of whether it was to occur virtually or in-person, although one parent and their child thought an in-person family meal session might be more awkward. One parent noted that while uncomfortable, the family meal holds a great deal of importance, even if it occurs virtually, as it enabled the therapist to witness first-hand components of mealtime that are triggering for their child and helped in empowering parents to persevere through meal challenges. Parents also made remarks about having to weigh their child at the beginning of each session, as required in FBT-V. Most found that this component was difficult but stated that it became easier as the sessions progressed and recognized the importance of their child being exposed to their weight for recovery. One parent stated:

*“That was a little strange,* [obtaining and] *looking at the weight and telling* [patient] *the numbers. But then we got used to that because I guess you need to normalize them looking at numbers and scales and have to be comfortable with their weight…that was hard at first, but then it got better.”* (Site 4).

#### Suggestions for improvement

Four of the five families strongly suggested hybrid models of FBT in the future—consisting of a mixture of in-person and virtual sessions. Family members proposed having at minimum the first few sessions occurring in-person and then switching to virtual delivery, facilitating a balance of convenience and rapport. One parent stated:

*“It would’ve been better, I think, if we at least on the initial visit with* [therapist] *that we could’ve met face-to-face so that we get to know each other better and more intimate in terms of discussing the issues that we had.”* (Site 3).

One sibling agreed, saying:

*“I feel like a mix would be good. It’s great for convenience, but I think that the first ones should be in-person so that they actually meet each other… Engaging with someone in-person the first time you meet them… it would’ve been more effective to immediately feel comfortable right? I think that being in-person for that,* [therapist] *would be able to read* [patient’s] *emotions better…just to get to know* [patient] *better.”* (Site 3).

Other suggestions made by families included having the patient choose whether to receive virtual or in-person treatment, and being offered alternative virtual platforms that families might be more familiar with, like Microsoft Teams or Skype for Business. Some families proposed changes to FBT more generally. These included increased one-on-one time between the patient and the therapist, an additional family meal, and a reduction in the frequency of sessions (e.g., bi-weekly instead of weekly). Regardless of these suggestions, all families reported that they would recommend FBT, delivered virtually or in-person (depending on the circumstances), to other families affected by eating disorders. One child expanded on this, saying:

“*I definitely would…it really helped me realize a lot of things. This is my first time really learning about eating disorders, so I didn’t really know a lot about them and how serious they are until this whole thing. And like learning a lot about it makes me really look at a lot of things and how beneficial this could be to other people who potentially don’t know what an eating disorder is and how serious they actually are.*” (Site 1).

## Discussion

To our knowledge, this is the first study to qualitatively evaluate the perspectives of teams and families with respect to virtually delivered FBT for pediatric eating disorders during the COVID-19 pandemic. Experiences of initial FBT-V implementation were captured through focus groups with teams consisting of therapists, medical practitioners, and program administrators, as well as focus groups with families. Based on our qualitative findings, FBT-V is acceptable and feasible among teams delivering and families receiving the treatment, however suggestions for improvement were made. There was also a clear preference from families for in-person or hybrid models of treatment.

With respect to its advantages, teams acknowledged being able to treat more patients virtually than in-person, whereas families commented on the convenience, comfort, and cost-effectiveness of FBT-V. Therapists and families recognized technical difficulties as a disadvantage of FBT-V. Therapists and families differed in their perceptions of the therapeutic connection. Families described missing a strong connection to their therapist that they believed would have occurred if they met in-person, while one therapist felt that they became better acquainted with family dynamics by being virtually invited into their home. When recalling the task of weighing their child during each session, parents stated that this was difficult, yet it became easier over the course of the study, and like therapists, acknowledged that exposing their child to their weight was important for recovery. Families explained that while impactful, the family meal session was uncomfortable and awkward, and they presumed that this would be the case whether it occurred in-person or virtually. Nonetheless, families recognized the importance of FBT, as all families reported that they would recommend this treatment, delivered virtually or in-person (dependent on the circumstances), to other families living with eating disorders.

Regarding therapist perceptions of virtual therapy, other research mirrors some of our findings. A recent mixed methods study involving mental health clinicians delivering virtual care during the COVID-19 pandemic similarly had clinicians report virtual platforms as being easy to operate and recognized that virtual care enabled increased access to care for their patients/families; they also highlighted technical difficulties and trouble managing disruptions in their patients’ homes as strong challenges [25]. These clinicians also described that virtual care impacted their patient interactions including rapport building and managing confidentiality and privacy in their patients’ homes [25], Conversely, trouble developing therapeutic rapport was identified as a disadvantage by the patients and families in our study, and not identified as a challenge by our therapists. It is possible that during earlier stages of the pandemic when FBT-V was more novel, the individual with the eating disorder as well as their family not being in the physical presence of the therapist might have interfered with therapeutic alliance from the perspective of a therapist to a greater degree. However, as the pandemic persists, therapists may be becoming more accustomed to delivering therapy virtually, whereas this remains unusual for families, especially those that have never received eating disorder treatment before, and thus may have a stronger impact on their ability to connect with their therapist.

Considering additional familial views, a recent case study [26] involving three young women with eating disorders who received a virtual, home-based treatment model of care found perceptions of virtual treatment similar to our study. For example, some patients and families found the online intervention acceptable and assisted in improving their behaviours, but others noted several challenges that acted as barriers to effectiveness, such as feeling anxious and uncomfortable disclosing sensitive information online [26]. More generally, systematic review results related to patient views of virtual mental health care indicate patient satisfaction with psychotherapeutic interventions and therapeutic alliance that is comparable to in-person delivery [27]; however this review does not contain findings from the COVID-19 era, which might also have influenced some experiences with virtual care during our study. For example, participants in our study had no choice but to participate in FBT-V, as in-person FBT was not available due to COVID-19 restrictions. This might have created some frustration as there was no option for in-person treatment.

Our results suggest the importance of testing hybrid models of FBT in the future, as almost all family members expressed an interest in FBT that contains both in-person and virtual components. Alternatively, therapists and other implementation team members did not explicitly suggest hybrid models of FBT but did propose other suggestions for improved future FBT-V practice. These included proposing the option to record FBT-V sessions and reviewing these recordings with families at a later date to demonstrate progress, having family members log into the virtual platform on different devices, assessing a family’s suitability or motivation for FBT-V while emphasizing the expectations of virtual treatment (prior to its commencement) to ensure it would be a good fit, and advocating for FBT-V beyond the pandemic. It is important to note that focus groups amongst teams and families were occurring concurrently. Therefore, we were unable to inform therapists about the familial preference for in-person or hybrid models of treatment, as it was not yet concluded that most family members voiced this preference, and as a result, teams could not comment on this. Additionally, therapists were recruited to partake in our study aiming to examine our implementation approach of virtually delivered FBT, where a hybrid model of treatment was never part of our study protocol. Therefore, therapists may not have thought to comment on a hybrid model during their focus group, given their role in only delivering FBT-V for the purpose of this study. Even if therapist-provided feedback and suggestions (as stated above) are implemented into FBT-V, it is worthwhile to explore therapists’ preference for FBT delivery—whether it is in-person, virtual, or hybrid. Understanding the factors that influence preference amongst care providers may provide insight into how the nuanced relationship between therapists and pediatric eating disorder patients is affected by care delivery format.

Our study also lacked a direct comparison to what in-person FBT would entail during the COVID-19 era, including wearing masks and other personal protective equipment during sessions, remaining physically distanced from their therapist and possibly other family members, and the possibility of last-minute appointment cancellations due to someone unexpectedly being exposed, exhibiting symptoms, or testing positive for COVID-19. Given these safety measures required for any in-person treatment during this time, perhaps therapeutic rapport would not have been greater between each family and their therapist with in-person treatment. Furthermore, in some centres during the pandemic, only one parent was allowed to accompany their child to appointments, making in-person family therapy impossible. As a result, we believe future research should also be conducted that compares FBT-V to in-person FBT with COVID-19-related restrictions.

Findings from our study also support the need for greater examination of virtual adaptations of family therapy, including FBT. As new COVID-19 variants continue to emerge and potential “waves” create uncertainties for the future, it is safe to assume that virtual options for family therapy are likely here to stay, especially as families become accustomed to receiving services within their homes to some degree and mental health services recognize the cost savings of online therapy [28]. Furthermore, if family therapy continues to expand to be delivered virtually, this also creates an opportunity to test and examine virtually delivered training, supervision, and competence evaluation [29].

Notable strengths were apparent in our study. First, our study used rigorous qualitative methodology [22, 23], as we conducted separate focus groups with whole teams and families, respectively, to obtain a wide variety of perspectives pertaining to delivering and receiving FBT-V. Additionally, all families consented into our study remained in the study for its entire duration, and completed their focus group, and therefore no gaps in family data were present. Despite the challenges of staff turnover and illness, almost all staff participated in their respective end of study focus group (only three staff members did not attend). Our sample was also geographically diverse as we included sites and individuals from across Ontario, enabling representation from rural, urban, and remote settings.

Limitations to our study include its small sample size. Our research team made several attempts to increase the number of participating sites to have a larger sample size in our study, as we reached out to seven additional organizations. While expressing interest, all organizations declined, stating that they did not have the capacity to participate in research due to increased clinical caseloads, staff burnout, and decreased staffing resulting from the COVID-19 pandemic. Although one of our sites had three participating therapists trying to recruit study patients, only one family for one therapist was enrolled into this study; family recruitment was especially challenging at this site, given staffing changes and shortages.

Another limitation of this study is that we assessed family perceptions after only four sessions of treatment, rather than at the end of a full course of FBT-V. Had perceptions been studied at the end of FBT-V, families might have recalled different feelings and attitudes towards virtual care. The lack of a comparison group in our study is another possible limitation. While treatment teams could draw on previous experience with in-person FBT to comment on the pros and cons of FBT-V, it might have been more challenging for families to comment on this as they had never experienced FBT in person. Lastly, despite acknowledging the psychological impact of the COVID-19 pandemic on both families affected by and staff treating eating disorders [1, 2], our study did not aim to evaluate or mitigate this impact amongst these populations. As a result, future research in this area is required, particularly related to understanding how virtually delivered treatment might affect this psychological impact created by the pandemic.

## Conclusions

While team and family perceptions of FBT-V were generally positive, indicating that FBT-V appears to be acceptable and feasible, some suggestions for improvement were identified. Therapist-reported benefits of FBT-V included being able to treat more patients and enabling a better understanding of the family dynamic by being virtually invited into the families’ homes. Families that received FBT-V felt it was convenient, cost-effective, and beneficial in contributing to improved eating disorder symptoms. However, therapists noted lacking the same level of control over virtual sessions compared to in-person care, due to distractions in homes of families. Most families voiced a preference for in-person treatment due to difficulties in building therapeutic rapport with their therapist in FBT-V and were especially interested in a hybrid model of FBT that involved some in-person and virtual components. Further research is required, such as examining hybrid models of FBT among this patient population.

## Supplementary Information

**Additional file 1.** End-of-Study Focus Group Guide: Therapists, Medical Practitioners, Program Administrators.

**Additional file 2.** End-of-Study Focus Group Guide: Families and Patients.

## Data Availability

De-identified data from this study are not available in a public archive. There is no analytic code associated with this study. Material used to conduct the study are not publicly available.

## Abbreviations

FBT: Family-based treatment
FT-AN: Family therapy for Anorexia Nervosa
FBT-V: Family-based treatment delivered via videoconferencing
