# Correction: A qualitative evaluation of team and family perceptions of family-based treatment delivered by videoconferencing (FBT-V) for adolescent Anorexia Nervosa during the COVID-19 pandemic

**DOI:** 10.1186/s40337-022-00714-7

**Published:** 2022-12-08

**Authors:** Jennifer Couturier, Danielle Pellegrini, Laura Grennan, Maria Nicula, Catherine Miller, Paul Agar, Cheryl Webb, Kristen Anderson, Melanie Barwick, Gina Dimitropoulos, Sheri Findlay, Melissa Kimber, Gail McVey, Rob Paularinne, Aylee Nelson, Karen DeGagne, Kerry Bourret, Shelley Restall, Jodi Rosner, Kim Hewitt-McVicker, Jessica Pereira, Martha McLeod, Caitlin Shipley, Sherri Miller, Ahmed Boachie, Marla Engelberg, Samantha Martin, Jennifer Holmes-Haronitis, James Lock

**Affiliations:** 1 McMaster University, Hamilton, ON Canada; 2 McMaster Children’s Hospital, 1200 Main St W, Hamilton, ON L8N 3Z5 Canada; 3 Canadian Mental Health Association – Waterloo Wellington, Kitchener, ON Canada; 4 Chicago Center for Evidence-Based Treatment, Chicago, IL USA; 5 University of Toronto, Toronto, ON Canada; 6 Research Institute, The Hospital for Sick Children, Toronto, ON Canada; 7 University of Calgary, Calgary, AB Canada; 8 University Health Network, Toronto, ON Canada; 9 St. Joseph’s Care Group, Thunder Bay, ON Canada; 10 Grand River Hospital, Kitchener, ON Canada; 11 Southlake Regional Health Centre, Newmarket, ON Canada; 12 North York General Hospital, North York, ON Canada; 13 Stanford University, Stanford, CA USA

**Correction to: Journal of Eating Disorders (2022) 10:111** 10.1186/s40337-022-00631-9

Following the publication of the original article [1], we were informed of an error in co-author Gina Dimitropoulos's name: the name was incorrectly presented as "Gina Dimitropoulous".

The correct name is included in the author list of this Correction and has been updated in the original article.
