# Supplementary material for: A qualitative evaluation of team and family perceptions of family-based treatment delivered by videoconferencing (FBT-V) for adolescent Anorexia Nervosa during the COVID-19 pandemic
Source: J Eat Disord. 2022 Jul 26;10:111. doi: 10.1186/s40337-022-00631-9 (PMC9321306; doi:10.1186/s40337-022-00631-9)
Supplement: Supplementary file 1 — Additional file 1. End-of-Study Focus Group Guide: Therapists, Medical Practitioners, Program Administrators. [file 40337_2022_631_MOESM1_ESM.docx]

**Supplemental File 1**

**End-of-Study Focus Group Guide: Therapists, Medical Practitioners, Program Administrators**

Introduction

*Thank you for devoting your time to the virtual Family-Based Treatment (FBT-V) project to date and for agreeing to speak with me today about your experiences as a participant. We are now in the final stage of data collection and your input today will be a valuable addition to our evaluation of this project. The purpose of this focus group is to reflect on the experience of implementing FBT-V within your treatment program over the last year, and to talk a little bit about your successes and challenges with that process.*

*I’d first like to ask you some questions about the process of implementing FBT-V into your clinical practice. These questions relate to the overall process including: the training workshop, formation of implementation teams at your program, as well as the biweekly Zoom Healthcare meetings with the research team.*

- Can you please describe for me what it was like for you to incorporate “*virtual Family Based Treatment (FBT-V)*” into your clinical practice?
- How does the way we supported you to bring FBT-V into your clinical practice differ from what you have tried in the past?
- What aspects of the implementation model would you like to see used on an ongoing basis (e.g., training workshops, supervision, implementation calls)?
- What aspects of the implementation process, would you like to have changed?
- If you compare the way we helped to bring FBT-V into your clinical practice to the usual method of bringing virtual interventions into practice, how was our implementation model different?
- Were there any barriers to implementing FBT-V that arose during the study?

*Now I would like to ask you some questions that relate specifically to the online FBT-V training workshop. I would like you to think back for a moment to the training workshop provided at the start of the study that involved Dr. James Lock and Mrs. Kristen Anderson.*

- What are your thoughts about the FBT-V training workshop that we provided?
- Was there anything about the workshop that you found particularly useful?
- Was there anything about the training workshop that you would have liked to have seen added, or could have done without?
- What did you think about the inclusion of multiple program sites at the same training event? Do you feel that there are any benefits to this approach? What about any drawbacks?
- Was it important to have a physician knowledgeable in FBT-V speak about how the medical role is rather different in the FBT-V model?
- **For the MD/NP specifically** ---As a physician/nurse practitioner, how important do you think it was for you to be present at this workshop in order to implement FBT-V successfully within your program?
- **For the administrator** ---- As an administrator how important do you think it was that you were present at this workshop in order to implement FBT-V successfully in your program?

***For Therapists*** *-- Now I would like you to think about the biweekly Zoom Healthcare meetings that focused on the FBT-V model and fidelity to the model – Clinical Consultation.*

- Thinking about these biweekly virtual meetings, to what extent did you find these meetings helpful in your use of the FBT-V model?
- What was most helpful about these meetings?
- Is there anything about these meetings, that now, thinking back, you would like to have changed?
- Did these meetings help you maintain fidelity to the FBT-V model?
- Did you find it helpful to have your fidelity to the FBT-V model rated?
- How did your practice of FBT-V change as a result of receiving training and supervision?
- Do you think you will continue to use FBT-V within your clinical practice? Are there any aspects of FBT-V that you will drop?
- In what ways, if any, did team dynamics, impact your adoption and use of FBT-V within your clinical practice?
- In what ways, if any, did the different roles of individuals within the team impact your adoption and use of FBT-V within your clinical practice?
- Throughout the project, was your use of the FBT-V model also supported or monitored by other means in your program; for example, through peer supervision?
- Would you have liked additional supervision support?
- Would you have preferred to have individual supervision as opposed to group supervision (not applicable if only one therapist on the team) – or if individual supervision was provided, would they have preferred group supervision?

*Whole Implementation Team- I would now like to take a moment to ask you about the implementation team at your program. This team was formed at the start of the study and involved a lead therapist, medical practitioner, and an administrator.*

- To what extent do you think the implementation team at your program supported your adoption and use of FBT-V within your clinical practice?
- What sorts of things did the implementation team do that you felt were helpful?
- What sorts of things do you wish the implementation team did differently?

*Whole Team - I would now like you to think about the biweekly Zoom Healthcare implementation calls with our research team (these are the calls that involved the MD/NP, administrator and lead therapist).*

- To what extent did you find these calls helpful in your adoption of the FBT-V model within your program?
- What was most helpful about these calls?
- Is there anything about these implementation calls, that now thinking back, you would like to have changed?
- Would you have liked additional support?
- In what ways, if any, did team dynamics, impact your adoption and use of FBT-V within your program?
- Can you comment on how specific program characteristics [for example: size, staff communication, staff experience, team culture] may have impacted the application of the FBT-V model into your program?
- **To the NP/MD** --- Did your usual medical role change with the adoption of FBT-V in your program?
- How did this impact you?
- What support would you have preferred/needed from an implementation team standpoint that would have helped in the use of FBT-V in your program?
- If you think about how we supported clinician’s use of the FBT-V model in practice, what are your thoughts about the supervision and fidelity checking process that we provided?
- Have there been discussions within your implementation team about how to sustain fidelity of FBT-V after the project ends? What are your thoughts about how to do this?

*And now just a couple of general questions on the project to end our focus group.*

- Are there any aspects of the way we rolled-out and supported the project that would make you more or less likely to continue to apply FBT-V; and other virtually delivered evidence-based treatments more generally, in the future?
- To what extent did the FBT-V model align with your program’s vision and mandate?
- Can you comment on aspects such as timing of the implementation and the content of the model?
- Are there any aspects of the way we rolled-out and supported the project that would make you more or less likely to continue to apply FBT-V; and other virtually delivered evidence-based treatments more generally, in the future?
- Would you like to add anything else?

*Thank you for participating in this focus group. Your participation has been very much appreciated. If you have any more questions please feel free to email me.*
