# Supplementary material for: A qualitative evaluation of team and family perceptions of family-based treatment delivered by videoconferencing (FBT-V) for adolescent Anorexia Nervosa during the COVID-19 pandemic
Source: J Eat Disord. 2022 Jul 26;10:111. doi: 10.1186/s40337-022-00631-9 (PMC9321306; doi:10.1186/s40337-022-00631-9)
Supplement: Supplementary file 2 — Additional file 2. End-of-Study Focus Group Guide: Families and Patients. [file 40337_2022_631_MOESM2_ESM.docx]

**Supplemental File 2**

**End-of-Study Focus Group Guide: Families and Patients**

Introduction

*Thank you for devoting your time to the virtual Family-Based Treatment (FBT-V) project to date and for agreeing to speak with me today about your experiences as participants. We are now in the final stage of data collection and your input today will be a valuable addition to our evaluation of this project. The purpose of this focus group is to reflect on your experiences of FBT-V, and to talk about aspects of virtual care that you thought worked well or need improvement. It is estimated that this focus group will take a maximum of 60 minutes. Please know that you are not required to answer any questions that you would not like to answer. Only the research team will have access to qualitative results of this focus group – your therapist will not see the transcripts or watch this focus group recording.*

*I’d like to first ask you some questions about your experience with the Virtual Family Based Treatment Sessions (I will call it FBT-V from here). Please try your best to describe your experiences.*

**Do you have any initial thoughts or reflections of your experience in FBT-V?**

- What did you like most about the FBT-V sessions?
- What would you change about FBT-V sessions?
- What were the benefits you experienced from participating in this treatment (if any)?
- *In terms of potential benefits,*
  - Was there weight gain during FBT-V?
  - Was there any improvement in eating disorder symptoms?
  - *Only ask if relevant* - Did your number of binge and/or purge episodes per week change over the course of FBT-V (e.g. increase or decrease?)
- Did you feel relevant issues were addressed?
- What topics were covered during the sessions?
- How did you feel about your therapist?

*Next, I would like to ask about costs – including monetary costs or costs of your personal time – that you and your family might have experienced during this study and treatment.*

**Cost**

- Did your family have to make any sacrifices to attend the virtual sessions?
- Did you have to make any purchases to participate in the virtual treatment (e.g. did you have to buy a computer, headset, new internet plan?)
- Do you think virtual treatment is more cost-effective than in-person treatment (e.g. not having to pay for parking at the hospital, not having to drive to the hospital for sessions?)

*We are almost at the end of this interview, but first can you please describe your thoughts and experiences with the virtual format of the sessions?*

**Virtual Format**

- What are your thoughts about the virtual format?
  - Did you like Zoom Healthcare? Is there another videoconferencing platform you might have preferred?
- How did you feel about your sessions being recorded in Zoom, particularly the family meal session?
- How did you feel about the weighing portion of the session via Zoom?
- How do you think virtual delivery would compare to in-person delivery?
- Did you or your therapist experience any technical difficulties?
- Do you think the virtual format affected your relationship with your therapist?
- Do you think the virtual format affected the initial effectiveness of the treatment?
- Is there anything you liked about the virtual format for FBT?
- Is there anything in the virtual format for Family-Based Treatment that you would change?

*Before we conclude this interview, I am lastly going to ask you to please describe any final thoughts, experiences, or words that you would like to share about the study and FBT-V.*

**Final Thoughts**

- Overall, when you reflect back on the virtual Family-Based Treatment sessions, do you have any additional comments on how the experience was for your family or how it could be improved?
- Would you recommend this virtual treatment or virtual treatment in general to another family?
- Do you have any final thoughts you would like to share about your experience in the study and/or virtual Family-Based Treatment?

*Thank you for participating in this focus group. Your participation has been very much appreciated. If you have any more questions please feel free to email me.*
